# Effects of glucagon‐like PEPTIDE‐1 receptor agonists on incidence of hepatocellular carcinoma and liver decompensation in patients with diabetes: A systematic review and META‐analysis

**DOI:** 10.1111/eci.70000

**Published:** 2025-02-12

**Authors:** Andrea Pasta, Antonio Facciorusso, Maria Corina Plaz Torres, Edoardo G. Giannini, Rodolfo Sacco

**Affiliations:** ^1^ Gastroenterology Unit, Department of Internal Medicine University of Genoa Genoa Italy; ^2^ Gastroenterology Unit, Department of Experimental Medicine Università del Salento Lecce Italy; ^3^ Gastroenterology Unit, Department of Medical Sciences University of Foggia Foggia Italy

**Keywords:** diabetes, hepatocellular carcinoma, liver decompensation, metabolic‐associated steatotic liver disease, treatment

## Abstract

This systematic review and meta‐analysis evaluated the impact of glucagon‐like peptide‐1 receptor agonists (GLP‐1RAs) on hepatocellular carcinoma (HCC) and liver decompensation in patients with type 2 diabetes. Analysing over 641,377 patients, GLP‐1RA use was associated with a significant 58% reduction in HCC risk, particularly in patients with cirrhotis. While a trend towards reduced liver decompensation risk was observed, it was not statistically significant. These findings suggest a potential role for GLP‐1RAs in HCC risk stratification and prevention strategies.

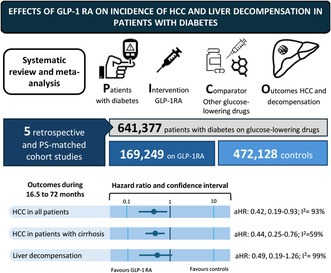

## 1 INTRODUCTION

Glucagon‐like peptide‐1 receptor agonists (GLP‐1RAs) are drugs approved in patients with type 2 diabetes (T2DM), or obesity, to control blood glucose and achieve weight loss.^1^ The presence of the Metabolic Syndrome, a condition characterized by overweight/obesity and a high prevalence of T2DM, is a major determinant of the occurrence of liver‐related events in patients with chronic liver disease, and T2DM is a well‐recognized risk factor for hepatocellular carcinoma (HCC) in these patients.^2^, ^3^ There is initial evidence that use of GLP‐1RAs may be associated with a decreased incidence of major liver‐related outcomes, such as liver decompensation and development of HCC, although the results of the various studies performed so far are inconsistent.^4^

Current evidence suggests that GLP1‐RAs might influence the progression of liver disease, either directly, through multiple activities such as regulation of glucose and lipid metabolism by modulating the activity of forkhead box protein O1 (FOX01), and improvement in liver anti‐oxidant capacity and metabolic function through activation of sirtuin‐1 (SIRT‐1), and indirectly by decreasing food intake and body weight.^4^

Given the increased risk of T2DM in patients with chronic liver disease of any aetiology, and the projected growth in the cases of metabolic dysfunction‐associated steatotic liver disease (MASLD)‐related HCCs in the near future, we deemed it of interest to assess whether, in patients with T2DM, the use of GLP‐1RAs was associated with a lower incidence of HCC and liver decompensation.^5^, ^6^ In order to do so, we carried out a systematic review with meta‐analysis of the existing literature on this subject.

## 2 METHODS

This meta‐analysis included comparative studies fulfilling the following inclusion criteria and PICO format:

- (P)atients, patients with diabetes undergoing antidiabetic therapy.
- (I)ntervention, patients on GLP‐1RA treatment.
- (C)omparator, patients treated with insulin or other glucose‐lowering drugs.
- (O)utcomes, main outcomes were the incidence of HCC and liver decompensation.

Case reports, review articles, and non‐comparative studies were excluded. Bibliographic research was conducted on PubMed, EMBASE, Cochrane Library and Google Scholar including all studies fulfilling inclusion criteria published until July 2024. The search was conducted by 2 study investigators (AF and AP) using the string: ((((((glp‐1) OR (glucagon‐like peptide‐1)) OR (semaglutide)) OR (dulaglutide)) OR (liraglutide)) AND (HCC)) AND (cirrhosis).

The quality of included studies was independently assessed by two authors (AP, MCPT) according to the Newcastle‐Ottawa scale for non‐randomized studies.^7^ Disagreements were solved by discussion and following a third opinion (AF).

Data on HCC occurrence and liver decompensation were pooled and compared through a random‐effects model based on DerSimonian and Laird test, and summary estimates were expressed in terms of hazard ratio (HR) with 95% Confidence Interval (95% CI). In order to partially obviate to bias due to the different follow up length among the studies and, within each study, between the two treatment arms in terms of several covariates (i.e. use of other medications, age, sex, race, family history of cancer, obesity, complications of T2DM, comorbidities) and to take into account not only the number of events but also their timing and the follow‐up of censored patients, we used adjusted HRs (aHRs). Chi‐square and *I* ^2^ tests were used for across studies comparison of the percentage of variability attributable to heterogeneity beyond chance. Probability of publication bias was not assessed due to the limited number of included studies. *p* < 0.05 for chi‐square test and *I* ^2^ <50% were interpreted as low‐level heterogeneity.

## 3 RESULTS

**FIGURE 1:**
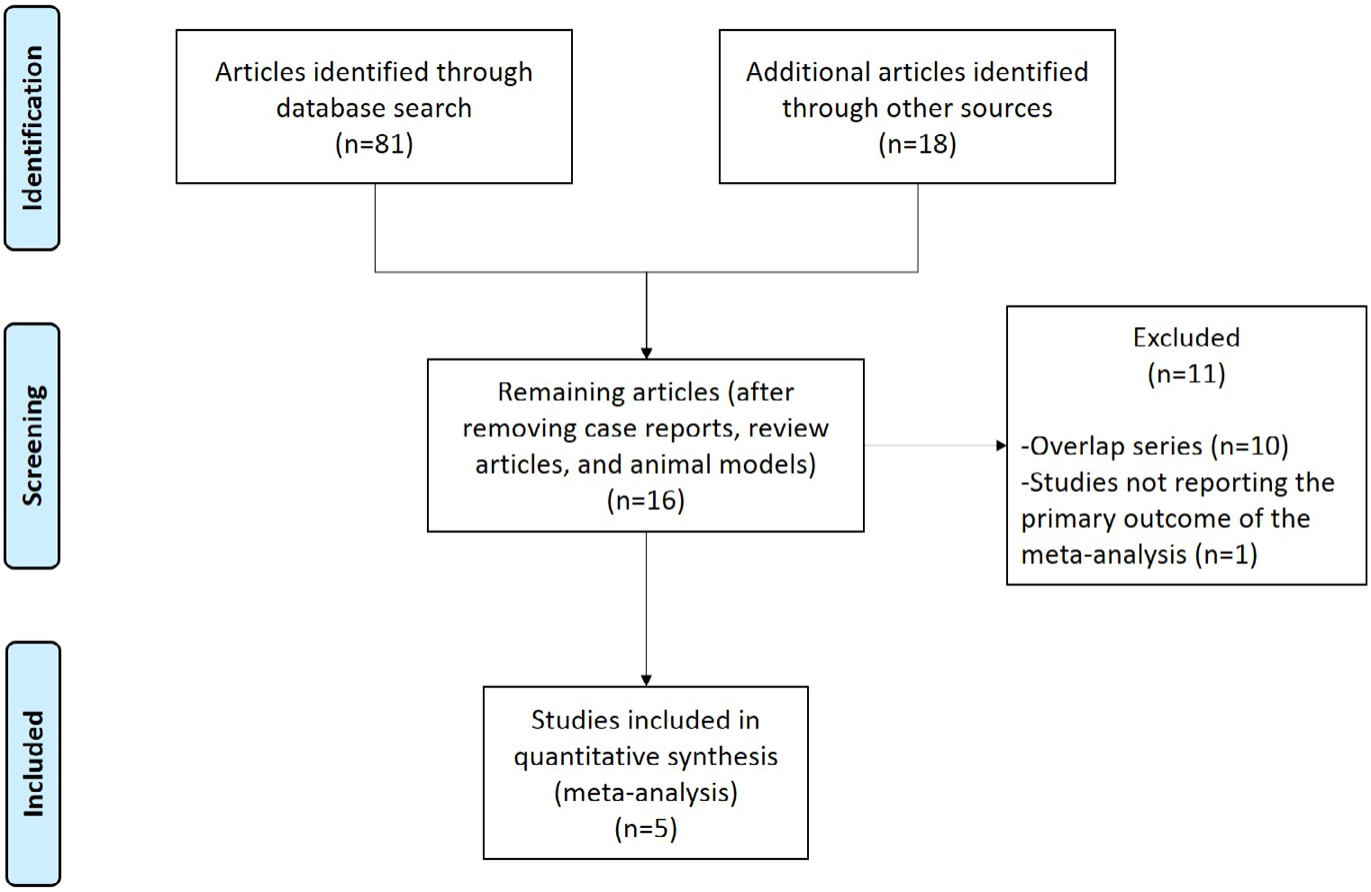
Flow chart of included studies.

Figure 1 reports the search strategy followed in this meta‐analysis: out of 99 studies initially identified, after preliminary exclusion of manuscripts not fulfilling inclusion criteria,^16^ potentially relevant articles were examined; among these studies, 10 overlap series and one study not reporting the incidence of HCC were further excluded. Finally, five studies including 169,249 patients on GLP‐1RA and 472,128 controls on insulin or other glucose‐lowering drugs were included in this meta‐analysis. All the studies included were propensity score‐matched, retrospective series (four were published in extenso, one in abstract form).^8^, ^9^, ^10^, ^11^, ^12^

The baseline clinical and demographic characteristics of the patients included in the meta‐analysis are shown in Table 1 and were well‐balanced between GLP‐1RA users and the control group. Aetiology of liver disease was mainly MASLD. Patients with cirrhosis were the totality in one study,^8^ a minor proportion in two other studies,^10^, ^11^ while they were not included in two further studies.^9^, ^12^ The methodological characteristics and the quality of the included articles are shown in Table 2. The recruitment period ranged from 2007 to 2020. Follow‐up length and duration of GLP‐1RA therapy ranged from 16.5 to 72 months.

**TABLE 1:** Main characteristics of the studies included in the meta‐analysis.

Author (reference)	Study period/Country/design/matching techniques	GLP1‐RA (exposure)	Duration of GLP1‐RA use (months)	GLP1‐RA (drug)	Age (years)	Sex (male)	Cirrhosis (presence)	Definition of hepatic decompensation
Elsaid^8^	2012–2020/USA/Retrospective cohort/OPSW	GLP‐1RA (+): 459 GLP‐1RA (−): 4837	16.5 (IQR 6.9–32.8)	NR	57 (51–61) 57 (51–61)	186 (40.6) 1964 (40.6)	0 (0) 0 (0)	Ascites, hepatorenal syndrome, spontaneous bacterial peritonitis, hepatic encephalopathy, oesophageal varices
Engstrom^9^	2007–2020 for Sweden and Denmark; 2010–2018 for Norway/Scandinavian Countries/ Retrospective cohort/PSM	GLP‐1RA (+): 91,479 GLP‐1RA (−): 244,004	36.0 (IQR 14.4–73.2)	Liraglutide: 81.3%; Exenatide: 10.4%; Semaglutide: 4.0%; Dulaglutide:3.5%; Lixisenatide: 0.8%	59.6 ± 10.7 59.8 ± 6.6	51,686 (56.5) 137,862 (56.5)	All patients/MASLD	Oesophageal varices, hepatorenal syndrome, portal hypertension, liver failure, liver transplantation
Kanwal^a^, ^10^	NR/USA/Retrospective cohort/PSW	GLP‐1RA (+): 23,670 GLP‐1RA (−): 169,646	20.0 (IQR 10.0–39.0)	Liraglutide: 13.0% Semaglutide: 70.0% Liraglutide + Semaglutide: 17.0%	NR	NR	3706 (15.7) 25,763 (15.2)	NA
Wang^11^	2013–2019/Global/ Retrospective cohort/ PSW	GLP‐1RA (+): 46,470 GLP‐1RA (−): 46,470	60.0	NR	56.1 ± 11.9 56.3 ± 14.7	19,424 (41.8) 18,727 (40.3)	418 (0.9) 372 (0.8)	Ascites, spontaneous bacterial peritonitis, hepatic encephalopathy, oesophageal varices
Yang^12^	2013–2018/Taiwan/ Retrospective cohort/PSM	GLP‐1RA (+): 7171 GLP‐1RA (−): 7171	72.0	Dulaglutide: 45.9%; Liraglutide: 54.1%	49.3 ± 12.4 49.1 ± 13.3	3686 (51.4) 3707 (51.7)	0 (0) 0 (0)	NA

*Note*: Data are reported as absolute numbers (percentages) or mean (± standard deviation or with range) or median (interquartile range).

Abbreviations: GLP‐1RA: Glucagon‐like peptide‐1 receptor agonists; MASLD: Metabolic dysfunction‐associated steatotic liver disease; OPSW: overlap propensity score weighting; PSM: propensity score matching; PSW: propensity score weighting.

^a^ Study published as conference abstract.

**TABLE 2:** Quality scoring for included articles using Newcastle‐Ottawa Scale for cohort studies.

	Selection			Comparability		Outcome			
Study (reference)	Representativeness of the exposed cohort	Selection of the non exposed cohort	Ascertainment of exposure	Demonstration that outcome of interest was not present at start of study	Comparability of cohorts on the basis of the design or analysis	Assessment of outcome	Was follow‐up long enough for outcomes to occur?	Adequacy of follow up of cohorts	Total/quality
Elsaid^8^	B*	A*	A*	A*	A*B*	B*	A*	A*	9/H
Engstrom^9^	A*	A*	A*	A*	A*B*	B*	A*	A*	9/H
Kanwal^10^	C	A*	A*	A*	A*	B*	A*	C	6/M
Wang^11^	A*	A*	A*	A*	A*B*	B*	A*	A*	9/H
Yang^12^	A*	A*	A*	A*	A*	B*	A*	A*	8/H

*Note*: Study quality assessment performed by means of Newcastle/Ottawa scale (asterisk represents if the respective criterion within the subsection was satisfied).

Abbreviations: H, high; L, low; M, moderate; U, unclear.

**FIGURE 2:**
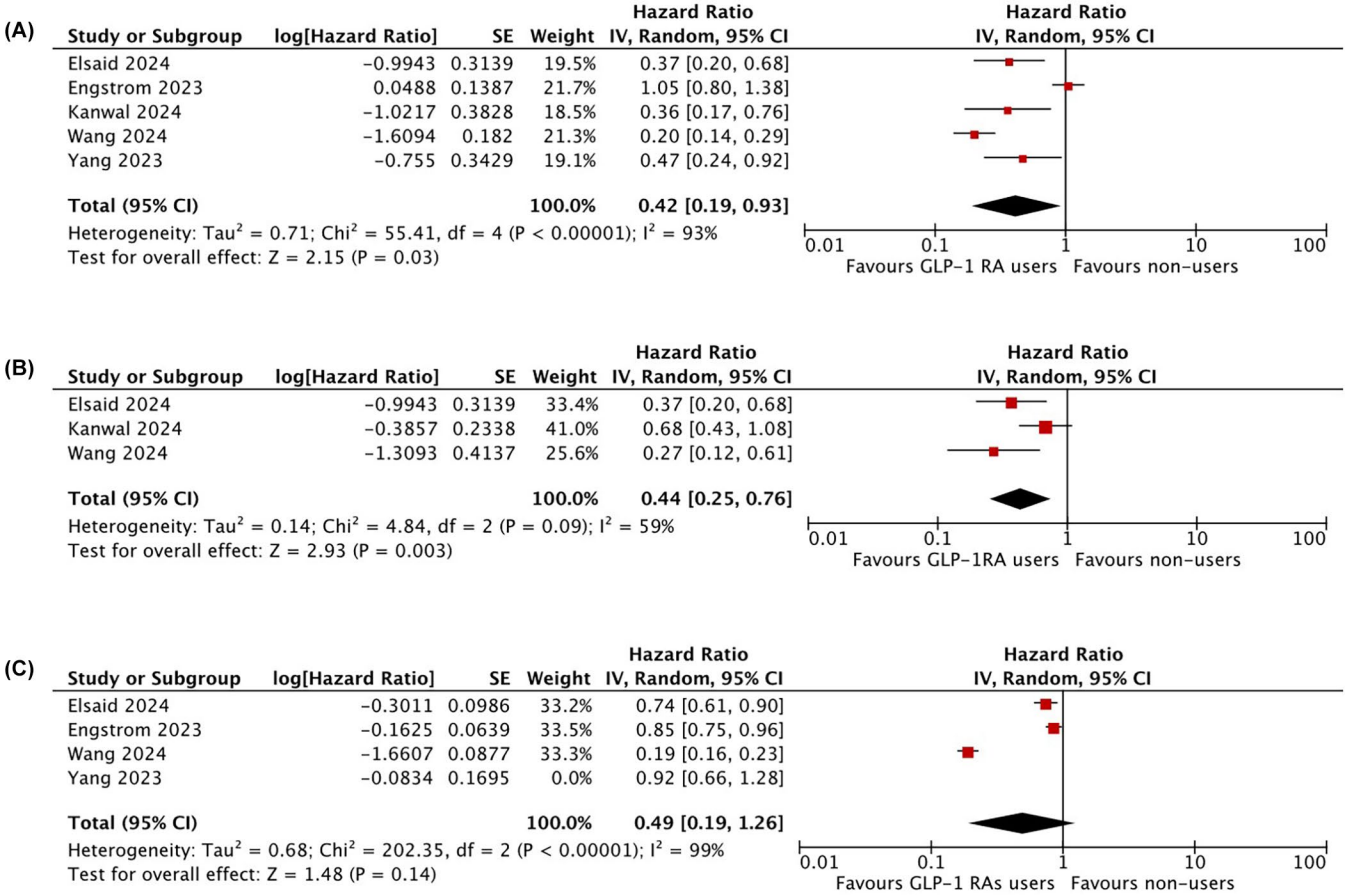
Forest plot assessing the adjusted hazard ratio (GLP‐1RAs users vs. non‐users) for the risk of: Occurrence of hepatocellular in all patients (A); occurrence of hepatocellular in the subset of patients with cirrhosis (B); occurrence of liver decompensation (C).

As reported in Figure 2A, the risk of HCC was significantly lower in patients on GLP‐1 RAs (aHR: .42, .19–.93), with high heterogeneity (*I* ^2^ = 93%, *p* <.00001) that was mainly due to the different magnitude of the effect rather than to its different direction. These findings were confirmed in the subset of patients with cirrhosis (aHR: .44, .25–.76), with a lower heterogeneity (*I* ^2^ = 59%, *p* = .09, Figure 2B). Shorter duration of GLP‐1 RA therapy (less than 20 months) did not influence the results (aHR .37, .23–.59) with no evidence of heterogeneity (*I* ^2^ = 0%, *p* =.96), while a more imprecise result was observed in the studies with longer duration of therapy (aHR .46, .14–1.50). Lastly, Figure 2C shows that the aHR for liver decompensation was .49 (.19–1.26), with evidence of high heterogeneity (*I* ^2^ = 99%, *p* <.00001).

## 4 DISCUSSION

In this meta‐analysis, that included more than 500,000 patients with T2DM treated with either GLP‐1RAs, insulin, or other glucose‐lowering drugs, we observed that treatment with GLP‐1RAs is associated with a significantly lower risk of developing HCC. Considering all patients, use of GLP‐1RAs determined a 58% decrease in the risk of occurrence of HCC as compared to the use of insulin or other glucose‐lowering drugs, independently of the presence of chronic liver disease. Noteworthy, these positive results were confirmed in patients at higher risk of developing HCC, such as patients with cirrhosis, where the use of GLP‐1RAs was associated with a 56% decrease in its incidence. Finally, these results were independent of the presence of potential confounding factors such as age, sex, race, family history of cancer, obesity, complications of T2DM, and comorbidities. These results may have a direct and immediate influence on the criteria that could be adopted to refine the population of patients with MASLD in need of surveillance for HCC. In fact, given this result and the large population with MASLD at risk of HCC, use of GLP‐1RA is worth being explored as a tool to stratify HCC risk, and thus to refine the population where surveillance for HCC may be cost‐effective.^13^

We also observed that use of GLP‐1RA was associated with a not significant decrease in the risk of liver decompensation. This finding may have several explanations. The inclusion of patients without cirrhosis in the studies considered might have diluted the effect on this endpoint, while the occurrence of HCC in patients with MASLD is not an inherent finding of patients with cirrhosis alone. Moreover, a recent phase II trial has shown that treatment with a GLP‐1RA was not associated with histological improvement—either fibrosis regression or necro‐inflammatory resolution—in patients with metabolic dysfunction‐associated steatohepatitis^14^ These finding suggests that GLP‐1RA might exert an indirect ‘chemopreventive’ effect by improving the conditions that enhance the risk of HCC, such as obesity and glycemic control, rather than a direct effect on the liver, also taking into account that the expression of GLP‐1 receptors in the liver is debated.^15^, ^16^

Quite recently, another similar meta‐analytic review article reached similar conclusions, although with a different degree of risk reduction in the development of HCC (HR .74, .56–.96), and with positive results regarding the risk of decompensation (HR .68, .65–.72).^17^ The slight difference in this latter outcome may be explained by the fact that some of the articles included in this meta‐analysis derived data from overlapping databases, an issue that we managed to exclude in our analysis. In particular, some of the studies included in the meta‐analysis by Costa Passos et al. were based on commercial claims datasets or other electronic health records networks, where lack of access to patient‐level data might render ascertainment of decompensation more prone to error.^17^ This notwithstanding, also our meta‐analysis disclosed a positive trend regarding the risk of decompensation (aHR .49, .19–1.26), though with high heterogeneity, and we feel that such a relevant issue merits to be assessed in dedicated, prospective studies based on patient‐level data.

A limitation of our analysis lies in the variability of study designs (e.g. matching techniques), study periods, geographic regions, and patient characteristics (e.g. age, presence of cirrhosis, different GLP‐1RAs, and duration of use), all of which contribute to heterogeneity and may affect the reliability of the meta‐analysis outcomes. Furthermore, the inherent limitations of retrospective designs and the inclusion of one study reported only in abstract form further weaken the robustness of the findings. Additionally, publication bias was not assessed due to the small number of studies, though its potential impact on the results should be acknowledged. Unfortunately, it was not possible to perform subgroup analyses or meta‐regression due to the small number of studies available.^18^ This limitation may compromise a more comprehensive exploration of the sources of heterogeneity and may potentially weaken the precision of the conclusions drawn from the analysis. Finally, the relatively short follow‐up period (16.5–72 months) likely underestimates the true risk of HCC development. Nevertheless, we believe that conducting a randomized controlled trial large enough and with a sufficiently long follow‐up period to capture the incidence of a relatively uncommon event is unlikely to be feasible.

On the other hand, this is a comprehensive meta‐analysis of over 500,000 patients with diabetes, demonstrating a significant 58% reduction in HCC risk with GLP‐1RA use, supported by robust methodology, adjusted analyses, and exclusion of overlapping datasets, providing valuable insights for HCC risk stratification.

In conclusion, we observed that the use of GLP‐1RAs in patients with T2DM is associated with a significant decreased risk of HCC, and we feel that this result might be exploited to stratify HCC surveillance in this growing population.

## AUTHOR CONTRIBUTIONS

Rodolfo Sacco, Antonio Facciorusso. and Edoardo G. Giannini: design of the study. Andrea Pasta, Antonio Facciorusso and Maria Corina Plaz Torres: data search and analysis of the results. Andrea Pasta, Antonio Facciorusso, Edoardo G. Giannini and Rodolfo Sacco writing draft. Maria Corina Plaz Torres, Edoardo G. Giannini and Rodolfo Sacco revision. All authors approved the final version to be submitted.

## FUNDING INFORMATION

The authors have nothing to report.

## CONFLICT OF INTEREST STATEMENT

None of the authors have any relevant financial disclosures.

## Data Availability

The data used in this work was generated exploiting data taken from published manuscripts. The data underlying this article will be shared on reasonable request to the corresponding author.
